# Respiratory sarcopenia screening in dialysis patients: cross-sectional and multicentre study protocol

**DOI:** 10.1186/s12882-023-03390-0

**Published:** 2024-01-29

**Authors:** Francini Porcher Andrade, Sheila Borges, César Alencar da Silva Filho, Taís Ferreira Martins, Heloíse Benvenutti, Júlia de Melo Cardoso de Freitas, Fernando Saldanha Thomé, Cristina Karohl, Gabriela Correa Souza, Graziella França Bernardelli Cipriano, Paula Maria Eidt Rovedder

**Affiliations:** 1 Ciências Pneumológicas Post-Graduation Programme, Federal University of Rio Grande do Sul (UFRGS), Rua Ramiro Barcelos, 2400 2º andar, Porto Alegre, RS, 90035-003 Rio Grande do Sul Brazil; 2 Hospital de Clínicas de Porto Alegre (HCPA), Porto Alegre, Rio Grande do Sul Brazil; 3 Research Center in Sports Sciences, Health Sciences and Human Development, University of Maia (CIDESD-UMAIA), Maia, Portugal; 4 Science and Technology in Health Programme, University of Brasília (UnB), Brasília, Distrito Federal, Brazil; 5 Sciences of Rehabilitation Post-Graduation Programme, University of Brasília (UnB), Brasilia, Distrito Federal Brazil; 6 Medical Sciences Focused on Endocrinology Post-Graduation Programme, Federal University of Rio Grande do Sul (UFRGS), Porto Alegre, Rio Grande do Sul Brazil; 7 Faculty of Medicine, Federal University of Rio Grande do Sul (UFRGS), Porto Alegre, Rio Grande do Sul Brazil; 8 Instituto de Doenças Renais (IDR), Porto Alegre, Rio Grande do Sul Brazil; 9 Graduate Program in Food, Nutrition and Health, Medical School, Federal University of Rio Grande do Sul, Porto Alegre, Rio Grande do Sul Brazil

**Keywords:** Chronic Kidney Disease, Dialysis, Sarcopenia, Respiratory sarcopenia, Pulmonary function

## Abstract

**Background:**

Respiratory sarcopenia is characterized by the weakness of respiratory muscles associated with sarcopenia due to aging or systemic diseases such as chronic kidney disease (CKD). Patients with CKD undergoing dialysis are particularly susceptible to respiratory muscle weakness caused by factors such as fluid overload and electrolyte imbalance. This weakness not only affects ventilation but also impairs oxygen uptake and delivery to muscle tissue, potentially leading to severe sarcopenia. Thus, the objective of this study is to conduct a respiratory sarcopenia screening in patients with CKD undergoing haemodialysis (HD) and peritoneal dialysis (PD).

**Methods:**

This is an observational, cross-sectional and multicentre study conducted between March 2023 and March 2025. The study was approved by the Research Ethics Committee at two centres. Sarcopenia diagnosis is determined based on low handgrip strength and amount of appendicular skeletal muscle mass, assessed through bioelectrical impedance analysis. Respiratory sarcopenia is diagnosed in patients with sarcopenia who have low inspiratory muscle strength, evaluated through a manovacuometry test. The severity of sarcopenia and respiratory sarcopenia is defined, respectively, by low physical performance (measured using the Short Physical Performance Battery and Timed-Up and Go test) and pulmonary performance (measured through spirometry). Thus, this study will include 81 patients undergoing dialysis (41 on HD and 40 on PD) from three participating centres.

**Discussion:**

The literature has been focused on respiratory function in CKD; however, the relationship with sarcopenia remains understudied. We believe that, similar to appendicular skeleton muscles, the axial skeleton muscles are also likely to weaken with the presence of chronic disease, such as CKD.

## Introduction

As well as the appendicular skeletal muscles, the respiratory muscles also undergo functional aging decline [1, 2]. Therefore, respiratory sarcopenia can be defined similarly to sarcopenia described by the Writing Group for The European Working Group on Sarcopenia in Older People 2 (EWGSOP2) [3]. Thus, it refers to the low strength of respiratory muscles associated with low quantity and quality muscle [4] mass due to aging or secondary to a systemic disease, particularly those that may trigger inflammatory processes such as chronic kidney disease (CKD) [3].

Respiratory muscle strength is a way to assess pulmonary function and can be evaluated through the manovacuometry test [5]. Pulmonary function decreases in patients with CKD, especially those undergoing haemodialysis (HD) [6], due to a combination of factors including the duration of CKD diagnosis, HD vintage, fluid overload, and electrolyte imbalance, which can lead to uremic myopathy [7–9]. We believe that fluid overload is the primary cause of decreased pulmonary compliance in patients undergoing HD, resulting in long-term inspiratory muscle overload and weakness.

Inspiratory muscle weakness impairs not only ventilation but also oxygen uptake and the delivery of oxygen needed for muscle tissue energy production [10, 11]. Gamboa et al. (2020) [12] demonstrated that muscle mitochondria are smaller in patients undergoing HD. Furthermore, dysregulation of mitochondrial biogenesis and function contributes to muscle loss, leading to sarcopenia and frailty [13]. Consequently, the development of sarcopenia in patients with CKD may stem from multiple factors, encompassing not only the health of appendicular muscles but also that of the respiratory and cardiovascular systems.

The maximal inspiratory pressure (MIP), obtained through the manovacuometry test, evaluates the diaphragm strength, external intercostal muscles, as well as the accessory inspiratory muscles such as pectoralis minor, scalene, and sternocleidomastoid [14]. Therefore, MIP can be considered one of the diagnostic criteria for respiratory sarcopenia since inspiration is an active process dependent on muscle contraction [15]. The American Thoracic Society and the European Respiratory Society have defined a MIP cut-off value of -80cmH_2_O for men and -70cmH_2_O for women as the minimum to exclude clinically significant inspiratory muscle weakness [14, 16]. Additionally, the manovacuometry test also evaluates maximal expiratory pressure (MEP), which reflects the strength of the abdominal muscles and other accessory expiratory muscles involved in active expiration. Therefore, all the muscles assessed in the manovacuometry test are part of the axial skeleton.

Pulmonary volumes and airflows are other measurements that reflect pulmonary function, specifically pulmonary performance, and are evaluated through the spirometry test [17]. Forced vital capacity (FVC) is the main outcome obtained from the spirometry test and measures the total air volume that can be rapidly and completely exhaled by the lungs in a single respiratory manoeuvre. In addition, it is one of the outcomes used to determine airflow restriction [13] and the normal value is defined as being above the 5th percentile limits (-1.645 z-scores) [18]. There is evidence supporting that impairment in pulmonary volumes and airflow in patients undergoing HD may result from the progressive loss of respiratory muscle strength due to fluid overload [8, 19]. Additionally, the HD vintage also appears to hurt pulmonary function, and the explanation is focused on the uremic milieu [20, 21].

According to the EWGSOP2 [3], low muscle strength is the primary parameter for identifying probable sarcopenia and serves as the most reliable measure of muscle function (i.e., muscle strength). However, the diagnosis of sarcopenia is confirmed by the presence of low muscle quantity or quality. Furthermore, the severity of sarcopenia is determined by a combination of low muscle strength, low muscle quantity or quality, and low physical performance [3]. In this context, low respiratory muscle strength, especially the inspiratory muscles, may diagnose respiratory sarcopenia in those patients who have confirmed diagnosis of sarcopenia [22]. Moreover, the severity of respiratory sarcopenia can be determined by low pulmonary volumes and airflows, i.e., by low pulmonary performance.

Therefore, the main objective of this study is to conduct a respiratory sarcopenia screening in patients with CKD undergoing dialysis. As secondary objectives we intend i) to evaluate the presence of sarcopenia, diagnosed by low handgrip strength and low appendicular skeletal muscle mass (SMM) in patients with CKD undergoing dialysis; ii) to determine the severity of sarcopenia through low physical performance in patients with CKD undergoing dialysis; and iii) to evaluate the presence of respiratory sarcopenia, diagnosed by low MIP in patients with CKD undergoing dialysis with confirmed sarcopenia; iv) to determine the severity of respiratory sarcopenia through low pulmonary performance in patients with CKD undergoing dialysis; and v) to evaluate the risk factors associated with respiratory sarcopenia and severe respiratory sarcopenia diagnosed in patients with CKD undergoing dialysis.

## Methods/design

### Study design, setting and participants

**Fig. 1:**
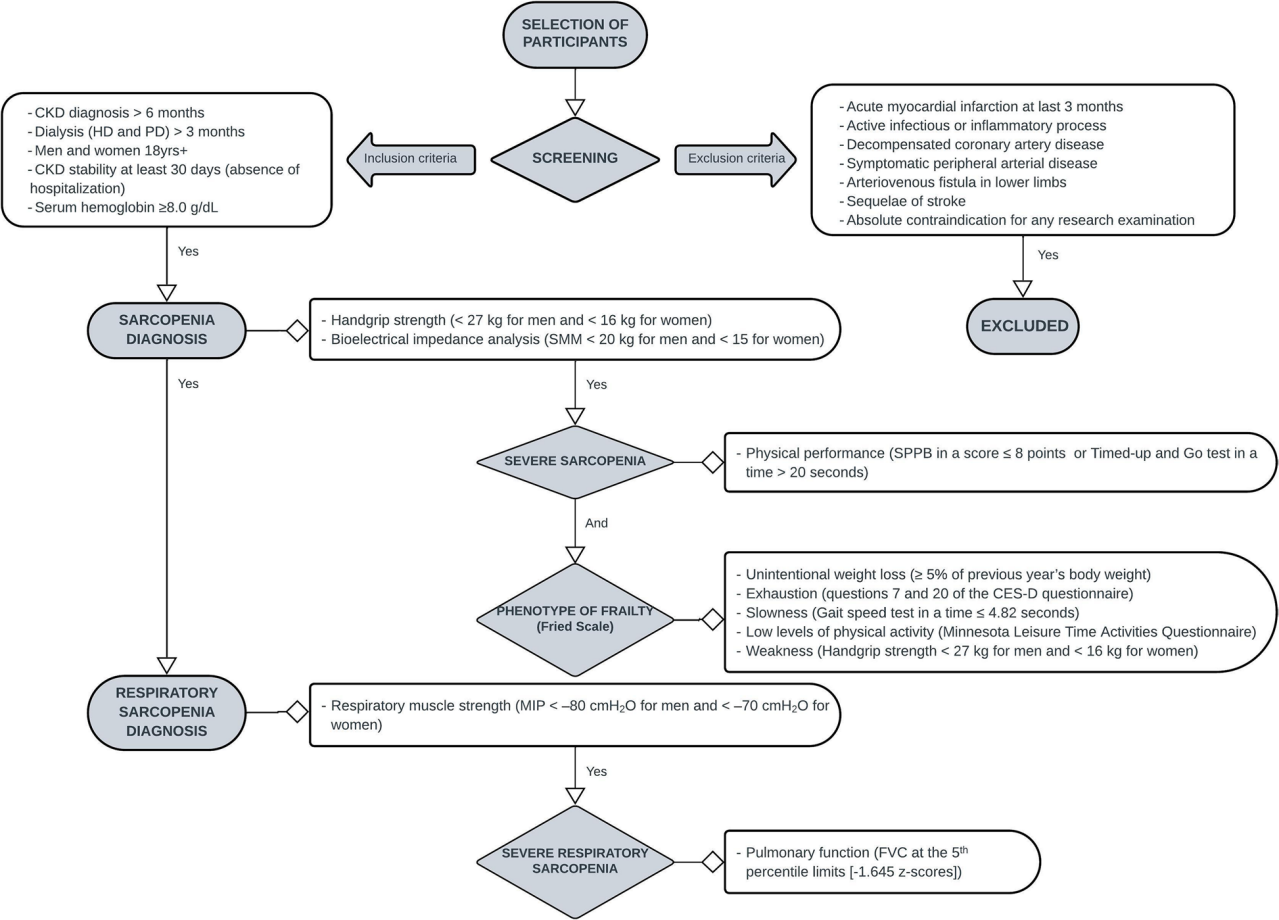
Study flowchart. SMM: skeletal muscle mass; SPPB: Short Physical Performance Battery; CED-D: Center of Epidemiological Studies Depression; MIP: maximal inspiratory pressure; FVC: forced vital capacity

This is an observational, cross-sectional and multicentre study, which performed a screening of respiratory sarcopenia between March 2023 and March 2025 in patients with CKD undergoing HD and peritoneal dialysis (PD) in the Nephrology Division of Hospital de Clínicas de Porto Alegre (HCPA), Instituto de Doenças Renais – IDR, both in Porto Alegre, Brazil, and the Nephrology Division of Hospital Regional de Taguatinga, Brasília, Brazil. The study setting and the eligibility criteria (inclusion and exclusion) are shown in Fig. 1.

### Ethical issues

Ethics approval was granted by the Research Ethics Committee of the HCPA and Fundação de Ensino e Pesquisa em Ciências da Saúde – FEPECS, approval number CAAE 63997122.1.0000.5327 and CAAE 63997122.1.3001.555, respectively. Written informed consent is obtained from all study participants according to the Declaration of Helsinki.

The researchers will maintain the confidentiality of the clinical and demographic data of the patients, and the results will be disclosed in a grouped mode. Participants can withdraw from the study at any time without affecting their clinical care.

### Measures and instruments

Patients undergoing HD are invited to participate in the study during their HD session, and those undergoing PD are contacted by telephone prior to their monthly medical appointment in the nephrology division of the aforementioned centres. Before the telephone contact, the researchers (physical therapists and nutritionists) analyse the patient's medical records considering the eligibility criteria (inclusion and exclusion). These criteria are discussed with nephrologists, and patients who do not meet the inclusion criteria due to serum haemoglobin values (≥ 8.0 g/dL) are reevaluated in the following month. The research team defined this cut-off point for haemoglobin values to prevent the interference of severe anaemia in the accuracy of physical test results.

All included patients undergo the sarcopenia and respiratory sarcopenia screening as mentioned in Fig. 1, as well as secondary evaluations including the frailty phenotype assessed through the Fried Scale, disease severity measured by the Charlson Comorbidity Index (CCI), fat percentage determined by skinfold measurements (biceps, triceps, subscapular and suprailia), muscle mass evaluated through calf circumference, and blood profile analysed for haemoglobin, albumin, urea, and calcium serum levels.

Patients undergoing PD undergo the evaluations in a single moment following their monthly medical appointment, while patients on HD undergo the evaluations at three separate moments. The HD evaluations are divided into three moments as follows: (1) questionnaires that comprise the Fried Scale and CCI performed during the HD session; (2) After the first interdialytic day or preferably before the second HD session of the week, both pulmonary function tests, including manovacuometry and spirometry, as well as physical tests such as handgrip strength, the Short Physical Performance Battery (SPPB), and the Timed Up and Go (TUG) test, are conducted; and (3) bioelectrical impedance analysis (BIA), follow by skinfold measurements, and calf circumference assessments performed immediately after the second HD session of the week, with a 7-day washout period. Blood tests are obtained from the patient's medical records within less than 30 days after the start of evaluations.

The cut-off criteria for diagnosing sarcopenia and respiratory sarcopenia, including their severity, are described in Fig. 1.

### Main assessments

The main assessments consist of tests used to diagnose sarcopenia and respiratory sarcopenia, and these assessments follow a specific order: manovacuometry, grip strength, spirometry, SPPB, TUG test, and BIA.

### *Respiratory* muscle strength

Manovacuometry is performed to evaluate inspiratory muscle strength using a digital manovacuometer (Microhard, Porto Alegre, Brazil) to measure MIP. MEP is also assessed. MIP and MEP measurements are taken from residual volume and total lung capacity, with the subject seated and wearing a nose clip. The inspiratory or expiratory effort is sustained for at least 3 s. The first three manoeuvres are selected if the difference in MIP and MEP values does not exceed < 10% variability between effort [14]. The highest value will be considered for the study. The results are expressed as absolute values and as a percentage of predicted values, as determined by the equations of Neder et al. (1999) [5, 14], which take into account sex and age:

- For males: MIP (cmH_2_O) = -0.80 (age) + 155.3
- For females: MIP (cmH_2_O) = -0.49 (age) + 110.4

A low absolute MIP is used to diagnose the respiratory sarcopenia and values less than -80 cmH_2_O for men and -70 cmH_2_O for women are considered low inspiratory muscle strength [14].

### Handgrip *strength*

The measurement of handgrip strength is conducted using a handheld hydraulic dynamometer (Saehan Corporation, South Korea in Porto Alegre centres and JAMAR®, Asimow Engineering, USA in Brasília centre), following the criteria established by the American Society of Hand Therapists [23]. During the measurement, patients must be seated with their elbow at a 90° angle, unsupported and their forearm in a neutral position for prone/supination. The wrist position could vary from 0° to 30° of extension and 0° to 15° of ulnar deviation. The feet are required to be firmly on the floor and the back support on the chair. Patients are instructed to squeeze the dynamometer as fast and hard as possible upon command [23]. Isometric contractions are performed on the non-arteriovenous fistula limb. The recorded values are observed in kilograms-force (KgF). Three repetitions are performed on each limb, with 60-s intervals between repetitions, and the average value is registered [24]. According to the EWGSOP2, low handgrip strength is defined as < 27 kg for men and < 16 kg for women. This criterion is used as a diagnosis of sarcopenia [3].

### Pulmonary performance

Spirometry is performed to assess pulmonary volumes and airflows using the spirometer Datospir MICRO (Sibelmed, Barcelona, Spain). The measurements obtained include FVC, forced expiratory volume in 1 s (FEV_1_), and peak expiratory flow (PEF), expressed in both absolute and predicted values. The spirometry exam is conducted with the patient seated and wearing a nose clip, following the guidelines of American Thoracic Society (ATS) and European Respiratory Society Technical Statement [25]. The patient observes the examiner demonstrating the exam and then receives instructions and encouragement to perform a maximal inspiration, followed by a forceful exhalation and continued complete exhalation for up to 15 s. The first three manoeuvres are selected if the difference in FEV_1_ and FVC values does not exceed 0.100 L and the highest value will be considered for the study [25]. The 5^th^ percentile limits (-1.645 z-scores) for FVC are used as a criterion to identify patients with results below the expected values, i.e., results that fall below the predicted values based on the patient's age, height, and sex, indicating the presence of severe respiratory sarcopenia [18].

### Short physical performance battery

The SPPB consists of assessing gait speed, balance test, and sit-to-stand test. A score ranging from 0 to 4 is assigned to each task, and a total score of ≤ 8 points indicates poor physical performance according to established criteria [3, 26, 27].

- *Gait speed:* The 4-m usual walking speed test is used to measure the time taken to walk in a marked corridor at a normal pace. Researchers demonstrate the route to the patient, who may use their usual walking aid. The best performance is defined by a time ≤ 4.82 s, which scores four points in the SPPB score [27]. According to the EWGSOP2, a cutoff speed ≤ 0.8 m/s indicates severe sarcopenia [3].
- *Balance test:* Patients are instructed to maintain three different positions for 10 s each: (1) standing with their feet side-by-side, (2) standing with the heel of one foot beside the big toe of the other foot, and (3) standing with the heel of one foot directly in front of the other foot. If completed, the first and second positions score 1 point in the SPPB score, while the third position scores two points [28].
- *Sit-to-stand test:* The sit-to-stand test evaluates lower limb muscle strength, specifically the quadriceps muscle group. It measures the time required to rise and sit in a chair with arms crossed over the chest, for 30 s. A chair without arms is used, with a rigid seat, stabilized against the wall, and a height of 47 cm from the floor. The time taken to complete five repetitions is monitored, and the best performance is defined by a time duration ≤ 11.19 s, which scores four points in the SPPB score [27]. A cutoff time > 15 s in the 5-repetition sit-to-stand test is defined as low resistance, and according to the EWGSOP2, it is used as an indicator of severe sarcopenia [3].

### Timed-up and Go

For the TUG test, the patient is required to rise from a chair, walk three meters as quickly and safely as possible, turn around at a designated point, return to the chair, and sit down again. A stopwatch is started when the researcher says the word 'go' and stopped when the patient is fully seated with their back against the backrest. Patients are instructed not to talk during the test and to wear their regular footwear. If needed, they may use their usual walking aid [3, 27]. A training test is conducted initially, and the value from the second test is recorded. According to the EWGSOP2, a cutoff time exceeding 20 s is an indicator of severe sarcopenia [3].

### Bioelectrical impedance analysis

The evaluation of body composition is performed using bioimpedance analysis. In the Porto Alegre centres, the equipment used is Biodynamics Corp from the USA, while in the Brasília centre, the Fresenius Medical Care Body Composition Monitor from Germany is utilized. Two distal electrodes are positioned on the dorsal surfaces, one on the hand (close to the metacarpal and phalangeal joint) and one on the foot (close to the metatarsal and phalangeal joint). Additionally, two electrodes are positioned on the pisiform prominence of the wrist and between the medial and lateral malleolus of the ankle. A current of 800 µA at 50 kHz is introduced through the distal hand and foot electrodes, using anthropometric parameters, sex, and age for a single-frequency measurement [29].

Patients are required to lie in a dorsal decubitus position without metallic accessories, with arms slightly abducted and legs slightly apart. They are instructed to rest for three minutes before measurements. Furthermore, patients are advised to fast for two hours following their usual routine. It is important to note that BIA does not directly measure muscle mass but estimates it based on whole-body electrical conductivity. Measurements include total body water, fat-free mass, fat mass, body cell mass, extracellular mass, resistance, and reactance. Skeletal muscle mass is estimated using the formula proposed by Janssen et al. (2000) [30], which takes into account sex (0 for women or 1 for men), height (centimetres), age (years), and resistance measured in ohms:

$$SMM (kg)\hspace{0.17em}=\hspace{0.17em}[(height.2/resistance\hspace{0.17em}\times \hspace{0.17em}0.401)\hspace{0.17em}+\hspace{0.17em}(gender\hspace{0.17em}\times \hspace{0.17em}3.825)\hspace{0.17em}+\hspace{0.17em}(age\hspace{0.17em}\times \hspace{0.17em}0.071)]\hspace{0.17em}+\hspace{0.17em}5.102$$

According to the EWGSOP2, a low amount of muscle is defined as an SMM of less than 20 kg for men and less than 15 kg for women [3].

### Secondary evaluations

The secondary assessments consist in Fried Scale, CCI, skinfolds, calf circumference and blood tests.

### Fried scale

The Fried Scale is used to evaluate the phenotype of frailty and requires a combination of physical measures and questionnaires [31, 32]. It assesses five structured components related to unintentional weight loss, exhaustion, slowness, low levels of physical activity, and weakness. These components are defined as follows: (1) unintentional weight loss of at least 5% of previous year’s body weight, which is assessed through medical records; (2) self-reported exhaustion, evaluated using questions 7 and 20 of the Center for Epidemiological Studies Depression (CES-D) [33]; (3) slowness, evaluated measuring the gait speed scoring cut-off [27]; (4) low self-reported physical activity levels, determined using the Minnesota Leisure Time Activities Questionnaire to assess weekly energy expenditure in the last two weeks [34, 35]; and (5) low strength, evaluated through handgrip strength below an established threshold [3]. Participants meeting at least three of these components are classified as frail [31]. Among non-frail participants, those meeting one or two criteria are classified as possibly prefrail, while individuals not meeting any criteria are considered non-frail [31].

### Charlson comorbidity index

The index used includes 19 comorbidities, each assigned a score of one, two, three, or six [36]. Comorbidities receiving a score of one include myocardial infarction, congestive heart failure, peripheral vascular disease, cerebrovascular disease, dementia, chronic pulmonary disease, connective tissue disease, ulcer disease, mild liver disease, and diabetes. Comorbidities receiving a score of two include hemiplegia, moderate or severe renal disease, diabetes with end organ damage, tumour, leukaemia, and lymphoma. Moderate or severe liver disease is the comorbidity receiving a score of three, while malignant tumour/metastasis and acquired immunodeficiency syndrome receive a score of six [36, 37]. As recommended by Charlson et al. (1994) [36], for a combined age-comorbidity score, one additional point is added to the risk for each decade of age over 40. A higher Charlson comorbidity score indicates increased severity of the condition [36].

### Skinfolds

The skinfold thickness measurement is conducted in four areas, including the biceps, triceps, subscapular, and supra-iliac regions. The skinfold thickness is measured to the nearest millimetre, except for values ≤ 5 mm, which is taken to the nearest 0.5 mm. The measurements are performed on the non-arteriovenous fistula side of the body while the patient stands in a relaxed condition [38]. The instrument used for the measurements is the Lange Caliper (NutriActiva, China). The standard technique described by Durbin and Womersley (1974) is employed for measuring the skinfolds [38], with the exception that the subscapular skinfold is always taken at an angle of approximately 45º to the vertical, and the suprailia skinfold is positioned just above the iliac crest in the mid-axillary line.

### Calf circumference

Bilateral calf circumference is measured using a measuring tape at the point of greatest circumference in patients while they are standing [39]. Calf circumference measurement is a predictor of physical performance and survival, with a cut-off point of less than 31 cm [3].

### Blood tests

Serum values of haemoglobin, albumin, urea, and calcium are obtained from the patient's medical record.

### Bias

As a potential source of bias, we can highlight the distinct brands of equipment used by each centre that is part of the study, such as manovacuometry, spirometry, handgrip strength, and BIA.

### Statistical analysis plan

The data will be stored in the Microsoft Excel program and analysed using the Statistical Package for Social Sciences (SPSS), version 28.0. Statistical significance will be set at *p* < 0.05.

All data will undergo the Kolmogorov–Smirnov test to assess normality. Parametric quantitative data will be described using mean and standard deviation, non-parametric data will be described using median and interquartile range, and categorical data will be presented as frequencies and percentages.

To assess agreement between different evaluation instruments (brands) such as the handheld hydraulic dynamometer and BIA, Bland–Altman analysis will be performed. Correlation analysis will be used to determine the association between these instruments. Pearson's correlation test will be used for parametric data, while Spearman's correlation test will be used for non-parametric data. The strength of the correlation will be categorized as very low (*r* < 0.300), low (*r* = 0.300 to 0.500), moderate (*r* = 0.500 to 0.700), high (*r* = 0.700 to 0.900), or very high (*r* = 0.900 to 1.000) [40]. Additionally, the reproducibility and reliability of the values will be evaluated using the intraclass correlation coefficient (ICC) and Cronbach's Alpha. ICC values will be interpreted as low (< 0.40), moderate (0.40 to 0.75), or excellent (> 0.75) based on Fleiss' criteria [41] and Guidelines for Reporting Reliability and Agreement [42].

The prevalence of respiratory sarcopenia will be calculated by dividing the total number of cases by the total number of patients evaluated.

Patients will be divided into two groups based on the presence or absence of respiratory sarcopenia. Comparisons between groups will be performed using the Wilcoxon Mann–Whitney test for non-parametric data and the independent Student t-test for parametric data. Categorical data will be compared using the Chi-Square or Fisher's exact tests.

Logistic regression analysis will be conducted to explore factors associated with respiratory sarcopenia. Multiple logistic regression will be used for continuous factors, while binary logistic regression will be used for dichotomous factors. Age, gender, Kt/V, HD vintage, COVID-19 history, and smoking habit will be considered as associated factors.

## Discussion

Pulmonary function in CKD has received increasing attention in the literature. However, its relationship with sarcopenia remains underinvestigated. We believe that axial skeleton muscles, as well as the appendicular skeletal muscles [3], may experience impairment not only due to aging, but also due to the presence of chronic diseases, such as CKD, leading to reduced strength and physical performance. In CKD, fluid overload may alter respiratory mechanical properties, particularly reducing pulmonary compliance and the respiratory muscle activity [8, 43]. These factors, combined with changes already documented in the literature, such as transient hypoxemia recovered during HD [44], muscle mass loss [29], the uremic milieu [46], reduced force of diaphragmatic contraction [21], chronic inflammatory processes [45] and the high incidence of cardiovascular diseases [10], could exacerbate the extent of physical impairment in these patients. In doing so, we aim to enhance our understanding of the lung-kidney connection for improved future health outcomes in patients.

## Data Availability

The datasets used and/or analysed during the current study are available from the corresponding author on reasonable request.
